# Case for Open Access and the Current Situation with the University of California and Elsevier

**DOI:** 10.5811/westjem.2019.7.44615

**Published:** 2019-09-03

**Authors:** John P. Renaud

**Affiliations:** University of California, Irvine Libraries, Associate University Librarian for Research Resources, Irvine, California

In my own words:

In recent months, the University of California has gained significant attention for taking a strong stance in support of open access publishing of UC research as it negotiates new agreements with major journal publishers. Cambridge University Press has agreed, enthusiastically, to partner with UC in piloting this new model while, thus far, UC’s negotiations with Elsevier have resulted in a stalemate.

The University of California has a long history of supporting open access. The UC Academic Senate adopted an open access policy in 2013, with the goal of ensuring that future research articles authored by faculty at all 10 campuses of UC will be made available to the public at no charge. In 2015 the Presidential Open Access Policy expanded this stance to include all other authors who write scholarly articles while employed at the University of California.

As an academic librarian, my primary goal is to assist my community of faculty, students, researchers, and clinicians as they navigate the information life cycle, work to understand problems and create new knowledge. Open access is a business model that will ensure several outcomes in this regard. First, it allows the creators of information to maintain ownership of their most important asset, their intellectual property. Of equal importance, open access allows the public access to the information that they have paid to create. There is little research that does not have some form of public funding behind it, either explicitly through grants and other funding, or indirectly, through the labs, personnel, and other resources provided by state and federal funds. A third very important aspect of open access is that it provides a sustainable way for scholarly communication to take place.

This last point merits some amplification. I have been fortunate to have spent my career in well-supported, research-intensive institutions. I started my career after the severe budgetary challenges of the early 1990s, and when the 2008 financial crisis hit, the institution at which I worked had the political will and funding to protect library budgets. While there have been a few instances where licensing or other issues meant that we’ve been unable to provide access to a certain product, I have never had to decline a faculty or student request for information based on financial reasons. True, I have had to say “wait” – for the new fiscal year, for another license to expire, for special funding to be approved – but I have not had to say “never.”

But outside this rarefied and privileged world, information seekers and information professionals have to make dire choices all the time. I believe one of the most important aspects of my job is insulating my community from that harsh reality; indeed, a librarian’s job is to connect the users that we serve with all the resources that they need. Yet, for many libraries – as well as for many individuals and other organizations – the cost of accessing “pay-walled” articles, which still account for the vast majority of the scientific literature, is simply unaffordable.

While some might cynically ask why folks not directly engaged in the research enterprise might need access to certain highly technical content, or why it is so crucial that this content be openly available, there are two answers. First, as alluded to above, as taxpayers everyone contributes to creating it, so everyone should be able to read it. A second, more subtle answer is suggested by a caller to a National Public Radio program focused on the UC/Elsevier negotiations. The caller was a community physician and she lamented the fact that paywalls often kept her from content that would assist her in patient care and professional development. So, the conversation is a bit more nuanced in that regard: It’s not just about “the average people on the street” but about the folks who need information to do their best to help them.

For all the rhetoric about “transformative” scholarly communication in the University of California’s negotiations with Elsevier, the propositions being raised by the University are fundamentally conservative. We want UC authors to determine where and how they will share the intellectual property that they create, including where they will publish, what they will read, and what roles they will play in the editorial process.

The last few weeks have provided great assurance that the University of California going forward will have agreements based on open access principles, including with Elsevier. Norway has reached an open access deal with Elsevier and the University has reached an open access deal with another important publisher, Cambridge University Press. In these models, which work on the principles of “pay to publish,” costs are contained and risks mitigated for both institutions and publishers, which will create a sustainable and open scholarly ecosystem.

